# Melt fluxing to elevate the forming ability of Al-based bulk metallic glasses

**DOI:** 10.1038/s41598-017-11504-6

**Published:** 2017-09-08

**Authors:** B. J. Yang, W. Y. Lu, J. L. Zhang, J. Q. Wang, E. Ma

**Affiliations:** 1 Shenyang National Laboratory for Materials Science, Institute of Metal Research, Chinese Academy of Sciences, 72 Wenhua Road, Shenyang, 110016 China; 2 Department of Materials Science and Engineering, Johns Hopkins University, Baltimore, MD 21218 USA

## Abstract

Salt-fluxing treatment is an effective technique to improve the glass-forming ability (GFA) of bulk metallic glass (BMG)-forming melts, as demonstrated before in Pd- and Fe-based systems. However, it has been challenging to develop similar fluxing protocol for more reactive melts, such as Al-rich BMG-forming systems. Here we design new fluxing agents, from a thermodynamics perspective that takes into account combined effects of physical absorption and chemical absorption (reaction) between the fluxing agents and oxide inclusions. MgCl_2_-CaCl_2_ composite salts were selected, and their fluxing effects were systematically studied on an Al_86_Ni_6.75_Co_2.25_Y_3.25_La_1.75_ alloy, the best BMG-forming composition reported thus far for Al-rich alloy systems. The oxygen content was found to continuously decrease in the master alloy with increasing cycles of salt-fluxing treatment, with chlorate products on the surface suggesting concurrent physical absorption and chemical reaction. The fluxing treatment developed has enabled a record critical size (diameter) of 2.5 mm for Al-based BMGs. Our finding is thus an advance in developing highly desirable Al-based BMGs, and also provides guidance for designing processing protocol to produce larger-sized BMGs in other reactive systems.

## Introduction

Aluminum-based metallic glasses (Al-MGs) are arguably the most attractive among all metallic glasses, because of their exceptional specific strength, excellent corrosion resistance (better than conventional crystalline aluminum alloys), and relatively low cost^1–4^. Unfortunately, the glass-forming ability (GFA) of Al-rich alloys are notoriously low; the critical size for glass formation achieved so far is merely of the order of 1 mm, barely reaching the borderline of bulk metallic glasses (BMGs), despite relentless efforts since the discovery of melt-spun glassy ribbons in 1988^5–9^. New possibilities for potential applications would open up, such as much easier spray-forming of anti-corrosion Al-MG coatings^10^, if the GFA of Al-rich alloys can be increased significantly.

It is well recognized that the GFA of Al-rich alloys is highly sensitive to the chemical composition^11–14^. Thus, compositional control is of paramount importance. Oxygen is one of the elements that degrade the GFA of BMG-forming alloys^15^. Its adverse effect is generally attributed to heterogeneous crystal nucleation on tiny oxide inclusions inside the melt. To remove these oxide particles for better GFA, boron oxide fluxing^16^ is known to be an effective processing route, through trapping impurities inside the fluxing compounds that float to the surface of molten alloys. The most remarkable success was reported for Pd-based alloys, where a BMG with a diameter as large as 72 mm was achieved by cleaning the melt using B_2_O_3_ fluxing^17, 18^. B_2_O_3_ fluxing also had marked effects on Fe_40_Ni_40_P_14_B_6_ melts, resulting in a reduction of about three orders of magnitude in the critical cooling rate required for BMG formation and the widening of the supercooled liquid region by ~30 °C^19, 20^. However, the use of B_2_O_3_ fluxing has been limited to Pd-, Fe-B- and Pt- based systems, because it is only effective for alloys in which all the major constituent elements have an affinity to oxygen lower than boron^16, 19, 21, 22^. In the prototypical BMG-forming alloys, such as those based on Zr or Ti^23–26^, where the primary elements have high oxygen affinity, there is a pressing need for an effective fluxing agent. Recently, a method has been developed for removing oxygen from Zr_55_Cu_30_Al_10_Ni_5_ and Cu_47_Ti_34_Zr_11_Ni_8_ liquids by employing a molten salt^27^. This method is based on the high solubility for oxygen ions in molten calcium chloride, but its effect on pre-existing oxide inclusions has not been well explained. Therefore, it is of interest to clarify the purification mechanisms of chlorides for removing oxide impurities, and transfers this knowledge to more reactive systems, such as Al-based BMG-forming melts. This is the subject of this study.

For Al-TM (transition metal)-RE (rare earth) glass-forming alloys, the molten melts are prone to forming oxides, including an Al_2_O_3_ layer on melt surface, due to the strong affinity of Al with oxygen, although the solubility of oxygen dissolved in molten aluminum is very limited^28–30^. Along with the bursting of the initial protective layer due to turbulence, the amount of Al oxides entering the alloy melt continuously increases. Due to the poor wettability between these oxides and aluminum^31–35^, the inclusions agglomerate and easily become heterogeneous sites for the nucleation of crystals, reducing the GFA^36–38^. Also detrimental to GFA for the Al-TM-RE system is another type of oxides, the rare-earth oxides (*e.g*. Y_2_O_3_), which have more positive activity and lower wettability than alumina and easily distribute in the molten alloy as inclusions^39, 40^. Although the effect of a small amount of Y addition on GFA is uncertain and depends on Y concentration^41–44^, for Al-rich alloys the simultaneous presence of Al and Y markedly was found to promote the thermal stability of the amorphous oxide phase, enabling the growth of the amorphous oxide layer up to ~100 nm thickness in Al_87_Ni_3_Y_10_ MG^45^. The dense structure of Al-Y oxide due to the large difference in ionic radius between Al and Y ions also slows down the diffusion of oxygen ions through the amorphous oxide layer^46^. The yttrium oxide is thus surmised to have an adverse effect on the GFA of Al alloys.

**Figure 1:**
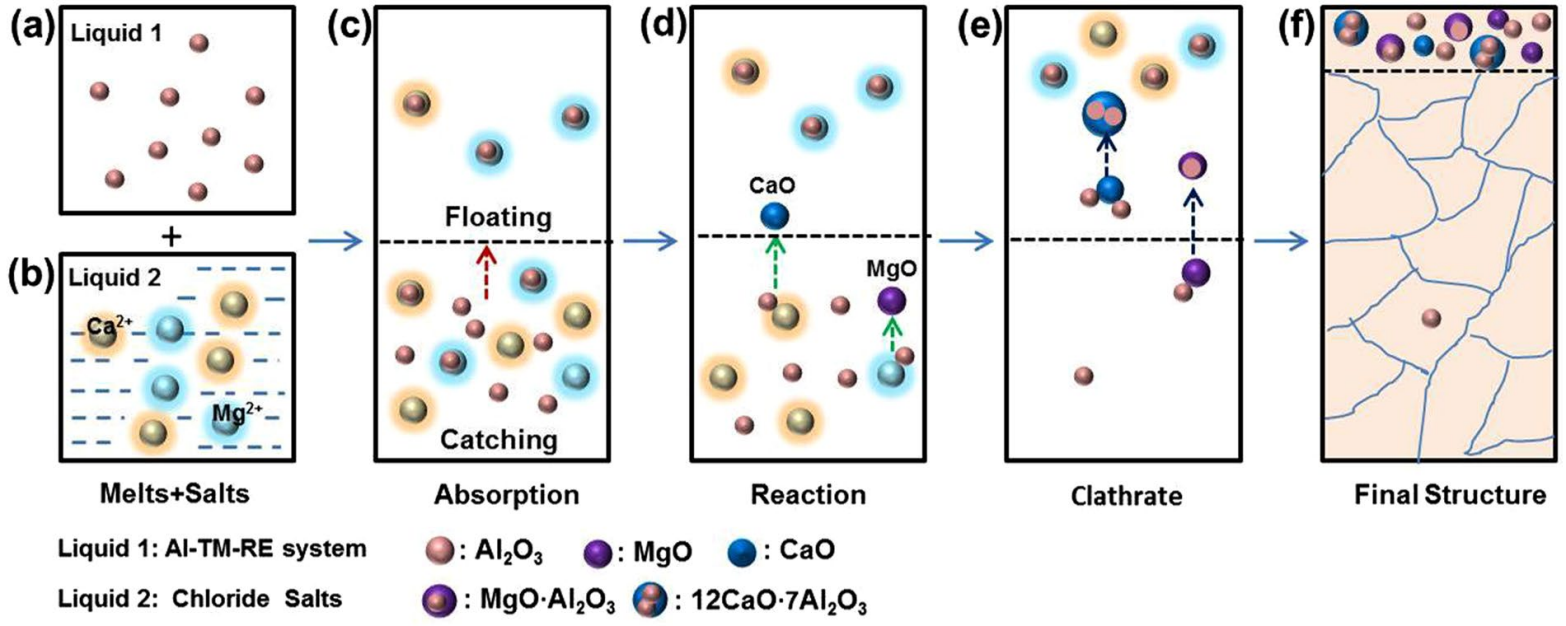
Schematic illustration of salt-fluxing to remove oxide inclusions in an Al-TM-RE alloy melt. (**a**) and (**b**) Al_2_O_3_ particles and fluxing salt in molten alloy, respectively. (**c**) Physical absorption, followed by the floating of the salt with caught Al_2_O_3_ particles due to the difference in mass density between the salt and the Al melt. (**d**) and (**e**) Chemical reaction and the subsequent clathrate compound formation between the oxides and fluxing salt, respectively. After the oxides have been absorbed into the salt, chemical reaction produces new oxides that could catch residual Al_2_O_3_ and form a larger-sized clathrate, eventually floating up to the melt surface. (**f**) The alloy microstructure and oxide distribution in the solidified master alloy.

Chloride salts are useful candidates as fluxing agents, since they have better wettability with the oxide inclusions than the Al alloy melt^47–49^. This would favor the separation of oxide inclusions from the melt and their absorption by the salts. The general idea of fluxing is schematically shown in Fig. 1. First of all, the chloride fluxing agents are low-melting-point, high fluidity compounds, and thus are capable of floating as molten salt on the surface of the molten Al alloy. Second, along the way of floating up to the top they can absorb oxide impurities and then agglomerate. Third, they can decompose to generate cationic (such as magnesium) ions, which are able to react with oxygen or oxide impurities in the aluminum melt, forming new low-density oxides (such as MgO) that float away. Fourth and finally, these new oxides would combine with residual oxide to form clathrate compounds such as MgO·Al_2_O_3_. This picks up additional oxide impurities in the molten melt^50^. The redistribution results in an oxygen-ridden surface layer on top of the melt, which can then be mechanically removed to leave behind a cleaner alloy.

We note that a single type of chloride salt may not be satisfactory. For example, NaCl has a quite strong fluidity in the melt, but does not present outstanding wettability and reactivity with the oxide inclusions. MgCl_2_ has sufficient wettability to absorb oxide inclusions, but its reactivity with aluminum oxide is relatively low. Therefore, a composite fluxing agent composed simultaneously of multiple chloride salts may be more successful.

Exactly which chloride salts should be selected is the first task we will be looking into in our work. Our salt selection will be based on thermodynamic considerations, analyzing the roles played by different chloride salts. We then investigate the performance of a selected fluxing agent in improving the GFA of Al-TM-RE alloy system. The quinary Al_86_Ni_6.75_Co_2.25_Y_3.25_La_1.75_ alloy will be used as the model, as this composition had the best GFA (1.5 mm in diameter, for Cu-mold casting) among all Al-rich alloys^9^. We report that a record size (diameter of 2.5 mm) BMG can be achieved at this composition using the fluxing protocol developed here.

## Design of fluxing agent

### Design based on combined effects of physical and chemical absorption

It is generally recognized that fluxing purification requires both effective physical absorption and chemical absorption. The physical absorption of the fluxing agent cleans up the melt through floatation of inclusions towards slag to remove heterogeneous nucleation sites. Its efficiency, for similar floatation velocity of inclusions, mainly depends upon the difference in wettability of the salt with the melt versus that with the oxide inclusions. Spontaneous physical absorption is a pre-requisite for the fluxing salt to be effective.

Chemical absorption, on the other hand, relies on chemical reaction at the interface between the fluxing agent and oxide inclusions. The reaction produces new oxides that have a lower interfacial energy with the melt and also possibly form clathrate compound with the residual aluminum oxide inclusions^51, 52^.

The key to design an effective melt fluxing agent lies in exploiting and balancing these purification effects. To select a salt, we used the following idea based on thermodynamic considerations. We start our design from the standpoint of the surface free energy change, the driving force behind the physical absorption. In the course of melt fluxing, oxide inclusions migrate from the molten metal to the salt agents. This process could be divided into three successive stages: (I) inclusion adhering to the boundary between the melt and the salt agent; (II) inclusion crossing over the interface between the melt and the salt agent; (III) inclusion leaving the phase interface and transferring into the interior of the salt agent. For oxide adhering to the interface of salt agent (stage I), the change of surface free energy, Δ*G*_1_, can be expressed as:

1 $${\rm{\Delta }}{G}_{1}=S[{\gamma }_{\mathrm{salt}/\mathrm{oxide}}-({\gamma }_{\mathrm{metal}/\mathrm{salt}}+{\gamma }_{\mathrm{metal}/\mathrm{oxide}})]$$

where *γ*_salt/oxide_, *γ*_metal/salt_ and *γ*_metal/oxide_ are the interfacial tension between the molten melt and the fluxing salt, molten melt and oxide, and oxide and fluxing salt, respectively. *S* is the surface area of oxide. When the oxide breaks away from the alloy melt and transfers into the molten salt (stage II and III), the change of surface free energy, Δ*G*_2_, is

2 $${\rm{\Delta }}{G}_{2}=S({\gamma }_{\mathrm{metal}/\mathrm{salt}}+{\gamma }_{\mathrm{salt}/\mathrm{oxide}}-{\gamma }_{\mathrm{metal}/\mathrm{oxide}})$$

Assuming that the oxide is not dissolved or deactivated by the molten salt, the surface free energy change on unit surface induced by physical adsorption, Δ*G*_p_^s^, would be the sum of these stages^53^, such that

3 $${\rm{\Delta }}{G}_{p}^{s}=({\rm{\Delta }}{G}_{1}+{\rm{\Delta }}{G}_{2})/2={\gamma }_{\mathrm{salt}/\mathrm{oxide}}-{\gamma }_{\mathrm{metal}/\mathrm{oxide}}$$

From Eq. (3), it is noted that the driving force for the oxide inclusions to go from the metallic melt into fluxing salt is related to not only the surface tension between oxide and fluxing salt but also that between the oxide and the melt. For the inclusions to spontaneously transfer into the fluxing salt, Δ*G*_p_^s^ should be negative. Hence a smaller *γ*_salt/oxide_ and lager *γ*_metal/oxide_ would entail a better efficiency of the fluxing salt to absorb the oxide inclusions. For a given oxide, its surface tension in Al melt is usually a constant. Thus, the reduction of surface free energy associated with physical adsorption is controlled by the surface tension between the oxide and the fluxing salt.

We next consider chemical absorption mediated by chemical reaction between the fluxing salt and oxide inclusions, which can accompany the physical absorption. The chemical interaction between the oxide (*e.g*. Al_2_O_3_) and fluxing salt (*e.g*., MgCl_2_) dissolved in liquid solution can be expressed as

4 $$3[{{\rm{MgCl}}}_{2}]+[{{\rm{Al}}}_{2}{{\rm{O}}}_{3}]=3[{\rm{MgO}}]+2[{{\rm{AlCl}}}_{3}]$$

The brackets denote that the elements are all in the liquid solution. Thus, the change in free energy per unit volume associated with chemical reaction, Δ*G*_*c*_^*v*^, can be calculated from^54^

5 $${\rm{\Delta }}{G}_{c}^{v}={\rm{\Delta }}{G}^{0}+RTln\frac{{\alpha }_{AlC{l}_{3}}^{2}+{\alpha }_{Mgo}^{3\,}}{{\alpha }_{MgC{l}_{2}}^{3}+{\alpha }_{Al2o3}^{1}}={\rm{\Delta }}{G}^{0}+RT\,\mathrm{ln}\,K$$

where *R* is the gas constant, *T* is the temperature (*e.g*. 1273 K), ΔG^0^ ${\triangle {\rm{G}}}^{0}$ is the free energy of standard state. *α*_MgCl2_, *α*_AlCl3_, *α*_MgO_ and α_Al2O3_ represent the activities of MgCl_2_, AlCl_3_, MgO and Al_2_O_3_, respectively, which can be approximated with their respective molar concentrations, giving *K*, the chemical equilibrium constant. Thus, a large negative Δ*G*_*c*_^*v*^ signals an easier reaction. The formation of different products such as MgO or clathrates in the melt could be determined by the equilibrium constant of the actual molten salt system^52^. This is consistent with West’s view^55^. In addition, the resulting product AlCl_3_ is volatile, so its impact on the melt is negligible^49^. The other product MgO has a density lower than Al_2_O_3_ and could combined with the latter forming a spinel structure MgO·Al_2_O_3_, which floats to the surface^56^.

One can also convert Δ*G*_*c*_^*v*^ to *ΔG*_*c*_^*s*^ (*i.e*. the surface free energy change per unit area) associated with chemical reaction, following the classic Butler’s equation^57, 58^. In this approach, it is assumed that the surface consisting of the outermost monolayer could considered to be an additional thermodynamic phase (*i.e*. in equilibrium with the bulk phase). Such an assumption has been shown to be valid in a number of experimental results^59–61^.

We now discuss the selection of fluxing salt in Al-rich alloy systems. Table 1 presents the related thermodynamic parameters and calculated values of *ΔG*_*p*_^*s*^ and *ΔG*_*c*_^*v*^ for five typical chlorides. For their combinations, we only considered two chlorides at their pseudo-binary eutectic composition, where a strong fluidity is expected at the low eutectic melting temperature^49, 62, 63^. This is because systems containing more components (*e.g*. three salts) have multiple eutectics with low eutectic temperatures, causing easy evaporation and difficulties to control composition. The ratio of the two chlorides salt is located at the eutectic composition (see Table 1). If there are multiple eutectics in the system, we used the one with the lowest eutectic temperature, as the low melting temperature of the mixture makes it easier to flow in the alloy melt and then easier to adsorb or dissolve of the inclusions.

**Table 1:** Thermodynamic data and calculated free energy change for different fluxing salts in molten Al alloy. A-B refers to the pseudobinary eutectic composition.

System (A-B)	Composition ratio (wt%)	*γ*_A-Al2O3_ (mN/m)	*γ*_B-Al2O3_ (mN/m)	*γ*_AB-Al2O3_ (mN/m)	Δ*G*_p_^s^ (mJ/m^2^)	*C*_A_	*C*_B_	*K*_A_	*K*_B_	Δ*G* ^1^_A_ (kJ/mol)	Δ*G* ^1^_B_ (kJ/mol)	Δ*G*_c_^v^ (kJ/mol)
NaCl		114.1	—	—	−31.4	—	—	—	—	—	—	0
KCl		98.3	—	—	−47.2	—	—	—	—	—	—	0
MgCl_2_		66.9	—	—	−78.6	1	—	5.39E+004	—	−115	—	−115
CaCl_2_		152	—	—	+6.5	1	—	2.97E+009	—	−230	—	−230
BaCl_2_		174.4	—	—	+28.9	1	—	5.38E+000	—	−178	—	−178
MgCl_2_-NaCl	55.3/44.7	66.9	114.1	84	−61.5	0.431	0.569	5.39E+004	—	−115	—	−49.5
MgCl_2_-CaCl_2_	43.5/56.5	66.9	152	93	−52.5	0.474	0.526	5.39E+004	2.97E+009	−115	−230	−175.5
MgCl_2_-BaCl_2_	37.8/62.2	66.9	174.4	92	−53.5	0.571	0.429	5.39E+004	5.38E+000	−115	−178	−142
MgCl_2_-KCl	64.2/35.8	66.9	98.3	79	−66.5	0.584	0.416	5.39E+004	—	−115	—	−67

*γ*_A-Al2O3_ (*γ*_B-Al2O3_) is the interfacial tension between salt A (salt B) and Al_2_O_3_. *γ*_AB-Al2O3_ is the average value of the interfacial tension in the binary salt system based on the relation: ${{\rm{\gamma }}}_{AB-Al2O3}=\frac{{\gamma }_{A-Al2O3}\,{\gamma }_{B-Al2O3}}{{\gamma }_{A-Al2O3}-({\gamma }_{A-Al2O3}-{\gamma }_{B-Al2O3})\ast x}$. The change of surface free energy due to physical absorption, ΔG_p_^s^, is *(γ*_AB-Al2O3_)-145.5, where 145.5 mN/m is the surface tension of Al_2_O_3_ in Al melt, and the density of Al_2_O_3_ is 4 g/cm^3^. The change of free energy in chemical reaction is Δ*G*_c_^v^ = Δ*G* ^1^_A_*C_A_ + ΔG^1^_B_*C_B_, where *C*_A_ and *C*_B_ are the molar concentrations of salt A and salt B at the pseudo-binary eutectic composition, respectively. Δ*G* ^1^_A_ and Δ*G* ^1^_B_ denote the changes of free energy due to chemical reactions between two kinds of salts (A or B) and Al_2_O_3_, respectively, where Δ*G* ^0^_MgCl2-Al2O3_, Δ*G* ^0^_CaCl2-Al2O3_ and Δ*G* ^0^_BaCl2-Al2O3_ is equal to −126 kJ/mol, −228 kJ/mol and −223 kJ/mol, respectively.

In Table 1, five chloride salts are considered. Among them, the first three, i.e., NaCl, KCl and MgCl_2,_ have negative *ΔG*_*p*_^*s*^, such that physical absorption would be spontaneous, whereas CaCl_2_ or BaCl_2_ show positive *ΔG*_*p*_^*s*^. MgCl_2_ has the largest negative value and is thus the best in this regard. In terms of chemical absorption, MgCl_2_, with its negative *ΔG*_*c*_^*v*^, also beats KCl and NaCl. Therefore, out of the three, we choose MgCl_2_ as the base of our flux system.

Next, we note that CaCl_2_ and BaCl_2_ show even more negative *ΔG*_*c*_^*v*^. This is particularly true for CaCl_2_, with its magnitude of *ΔG*_*c*_^*v*^ doubling that of MgCl_2_. CaCl_2_ may therefore be added into the MgCl_2_ base to further boost chemical reaction and absorption. Of course, this should be done at little expense of the physical absorption. As seen in Table 1, adding CaCl_2_ would sacrifice some physical absorption, but not by much: the *ΔG*_*p*_^*s*^ of MgCl_2_-CaCl_2_ remains negative and competitive with the MgCl_2_ base system. In the meantime, the large negative *ΔG*_*c*_^*v*^ of this combination stands out, setting it apart from other options in Table 1. As such, the best overall fluxing effectiveness is projected to reside with MgCl_2_-CaCl_2_, among these various salt systems.

### Fluxing of Al-TM-RE BMG-forming liquids

Having chosen the MgCl_2_-CaCl_2_ composite fluxing salt, we next consider its adaptability to Al-TM-RE alloys. Figure 1 depicts the three scenarios/effects: absorption alone, reaction alone, and the synergy between the two. In the Al-TM-RE system, Al_2_O_3_ and rare-earth oxides are present in the molten melt. Here we use Al_2_O_3_ for the purpose of illustration.

#### The physical absorption effect of chloride salts

As the MgCl_2_-CaCl_2_ composite fluxing salt floats up towards the top surface of the melt due to its lower mass density, it absorbs the Al_2_O_3_ inclusions and moves them to the melt surface, as schematically illustrated in Fig. 1(a~c). In the original Al melt, Al_2_O_3_ tends to agglomerate because they have very little wettability with molten aluminum. With the fluxing salts added, which have little solubility in the Al melt and do not react with Al, their favorable interfacial energies (see discussion above and Table 1) help separate the oxide and Al melt, and preferentially trap Al_2_O_3_ to eventually float up to the melt surface. This scenario helps the purification of the alloy melt.

#### The reaction effect of chloride salts

Figure 1(d) illustrates the role of chemical absorption in fluxing. Ca and Mg have higher affinity with oxygen compared with Al (*e.g*. the value of Gibbs energy of formation is −223 kJ/mol in the Al-Ca-O system, and −115 kJ/mol for Al-Mg-O system). This leads to the formation of new oxides such as CaO and MgO. Further, the clathrates between CaO (or MgO) and Al_2_O_3_ can form, such as 12CaO·7Al_2_O_3_ and MgO·Al_2_O_3_, which are thermodynamically stable and substantially increase the effect of absorption. Figure 1(d,e) schematically depict this process; the clathrates float up to the melt surface eventually. This is another scenario that enhances the purification.

The synergistic potency of physical absorption and chemical reaction is expected to provide an effective overall purification capacity. In other words, physical absorption and chemical reaction are taken into account at the same time, by simultaneously adding two fluxing salts. This is depicted in Fig. 1(a)~(f): the MgCl_2_-CaCl_2_ addition regulates both the interfacial tension and the interaction with oxide impurities in the melt, with a balanced potency of absorption and reaction. We therefore project that this “two-salts” composite combination would lead to a more desirable purification effect.

## Results

### Microstructure of the as-cast quinary alloy

**Figure 2:**
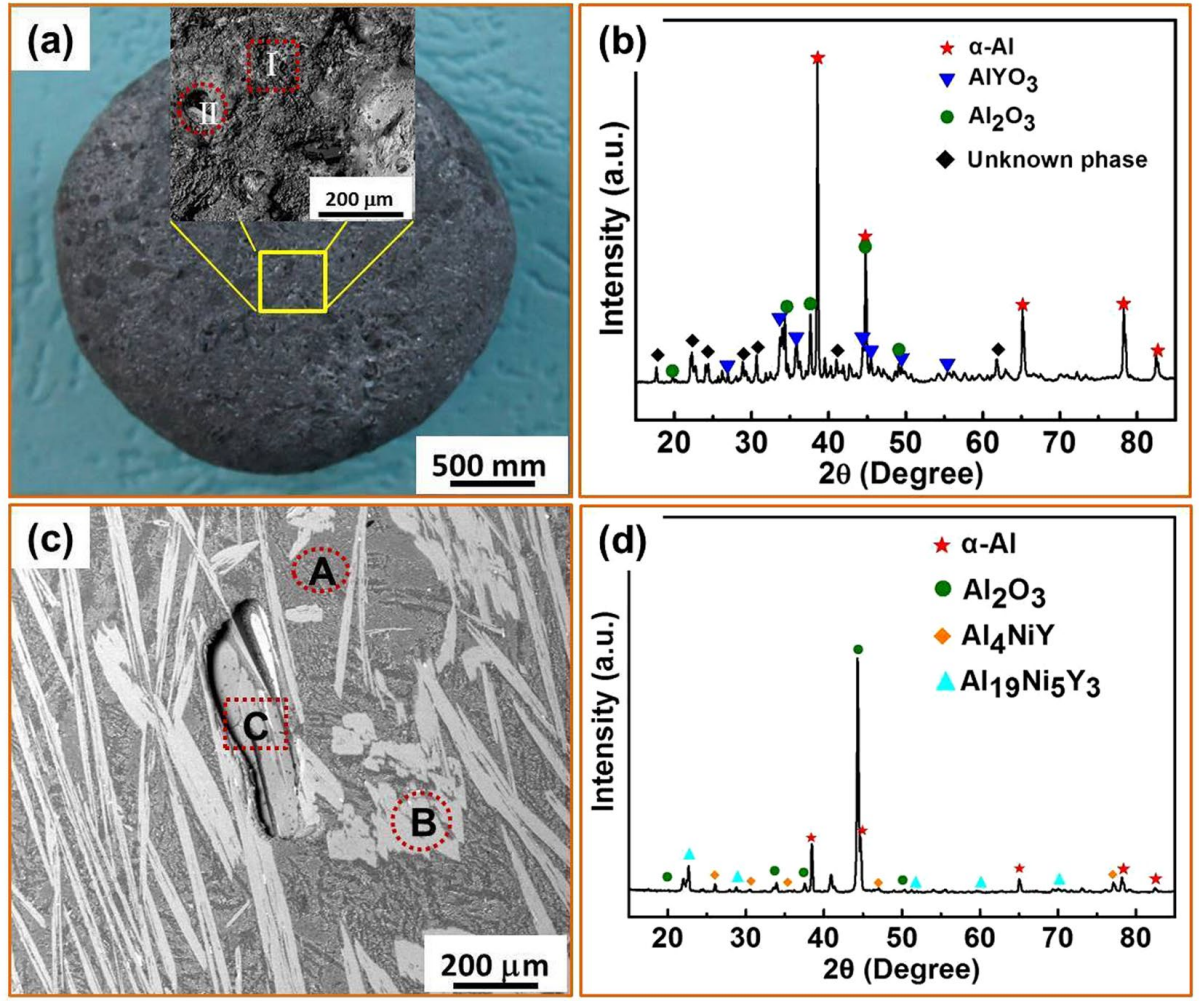
Microstructure of the Al_86_Ni_6.75_Co_2.25_Y_3.25_La_1.75_ master alloy without fluxing treatment. (**a**) SEM image of the sample surface with an enlarged view of the oxide layer taken from the yellow box region, showing a dark gray and loose oxidation surface layer; (**b**) XRD patterns from the surface; (**c**) SEM image of the cross section, the different regions within it are marked as: A-matrix, B-precipitate phase, C-a defect with second phase; (**d**) XRD scan of the cross section.

Figure 2 shows the structural characteristics of the as-cast Al_86_Ni_6.75_Co_2.25_Y_3.25_La_1.75_ master alloy without fluxing treatment. The typical appearance of this alloy is seen in the SEM micrograph of Fig. 2(a). The surface oxidation layer appears dark gray and relatively loose. The EDS analysis of the oxygen content in the surface layer (selected from yellow circled area in Fig. 2(a)) is shown: the oxygen content is in the range of 27~29 wt.% in two typical regions (I and II). Subsequently, we checked the structure of the crumbs of the surface layer obtained from carefully scraping off the whole surface oxide. As presented in Fig. 2(b), the XRD patterns showed that the surface layer is composed of α-Al, Al_2_O_3_, and AlYO_3,_ together with an unknown phase.

Figure 2(c) shows the interior structure of the master alloy and the distribution of oxygen content in the middle section of the cross-section. There are mainly three different areas: the Al matrix (region A), the precipitated phases (region B) and the casting defect area with lamellar large precipitated phase (region C). The oxygen concentration is around 1.06 wt % and 0.57 wt.% for region A and B, respectively. In region C, a number of precipitated oxides (~24.99 wt.% oxygen) are present with diameters of several micrometers. XRD of the master alloy, Fig. 2(d), shows α-Al, Al_4_NiY and Al_19_Ni_5_Y_3_ phases, along with some Al_2_O_3_. Combined with the oxygen concentration distribution measured in Fig. 2(c), we could infer that many Al_2_O_3_ inclusions randomly distribute in the matrix, and precipitated second phases and the Al_2_O_3_ inclusions are more concentrated in the defect-containing area than in other areas. Generally speaking, oxides have higher thermal and chemical stability than the melt itself in Al-rich alloys. Owing to the capillary adsorption of these oxides, an oxygen channel could form between the oxide layer and the melt. This induces the transmission and gathering of oxygen at the location of defect to form more oxide inclusions. Simultaneously, these channels can be blocked by other precipitated phases, finally forming the structure in region C in Fig. 2c.

### Microstructure of the quinary alloy after fluxing treatment

**Figure 3:**
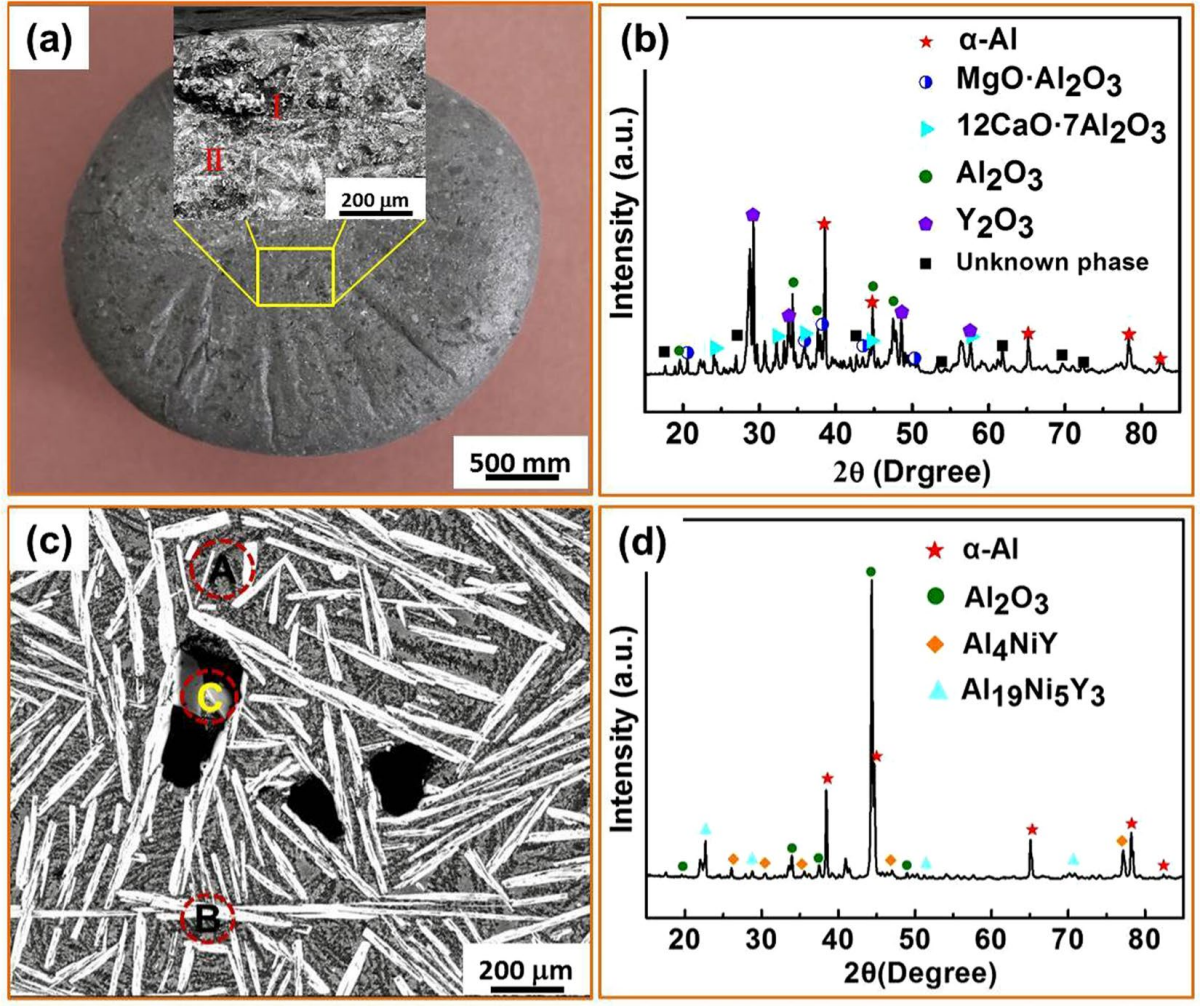
Microstructure of the Al_86_Ni_6.75_Co_2.25_Y_3.25_La_1.75_ master alloy after fluxing treatment for three heating-cooling cycles. (**a**) SEM image of the surface layer with an enlarged image taken from the yellow box region. (**b**) XRD pattern of the surface; (**c**) SEM image of the cross section under typical regions (**A**, **B** and **C**). (**d**) XRD pattern of the cross section.

Figure 3 depicts the structural features of the as-cast Al_86_Ni_6.75_Co_2.25_Y_3.25_La_1.75_ master alloy after three cycles of MgCl_2_-CaCl_2_ salt fluxing treatment. Compared with the untreated sample in Fig. 2(a), the treated master alloy appears brighter and the oxygen content of the sample surface tends to be lower (15~27 wt.% oxygen) in regions I and II. Figure 3(b) presents the XRD profile of the overall surface for the fluxing-treated master alloy. The structure is relatively complex, containing α-Al, Al_2_O_3_, Y_2_O_3_ and unknown phases, together with two composite oxide phases, MgO·Al_2_O_3_ and 12(CaO)·7(Al_2_O_3_). When compared with the phase constitution of alloy surface prior to fluxing treatment in Fig. 2(b), the appearance of Y_2_O_3_, MgO·Al_2_O_3_ and 12(CaO)·7(Al_2_O_3_) phases as well as the disappearance of AlYO_3_ phase could be attributed to the effect of salt fluxing.

In the sample interior, Fig. 3(c), the phases after fluxing treatment are still composed of α-Al, Al_4_NiY and Al_19_Ni_5_Y_3_ along with Al_2_O_3_. Nevertheless, the size of the second phase becomes smaller, and the oxygen content in the matrix (0.88 wt.%) and second phase (0.52 wt.%) are all much decreased (compare Figs 2c and 3c).

**Figure 4:**
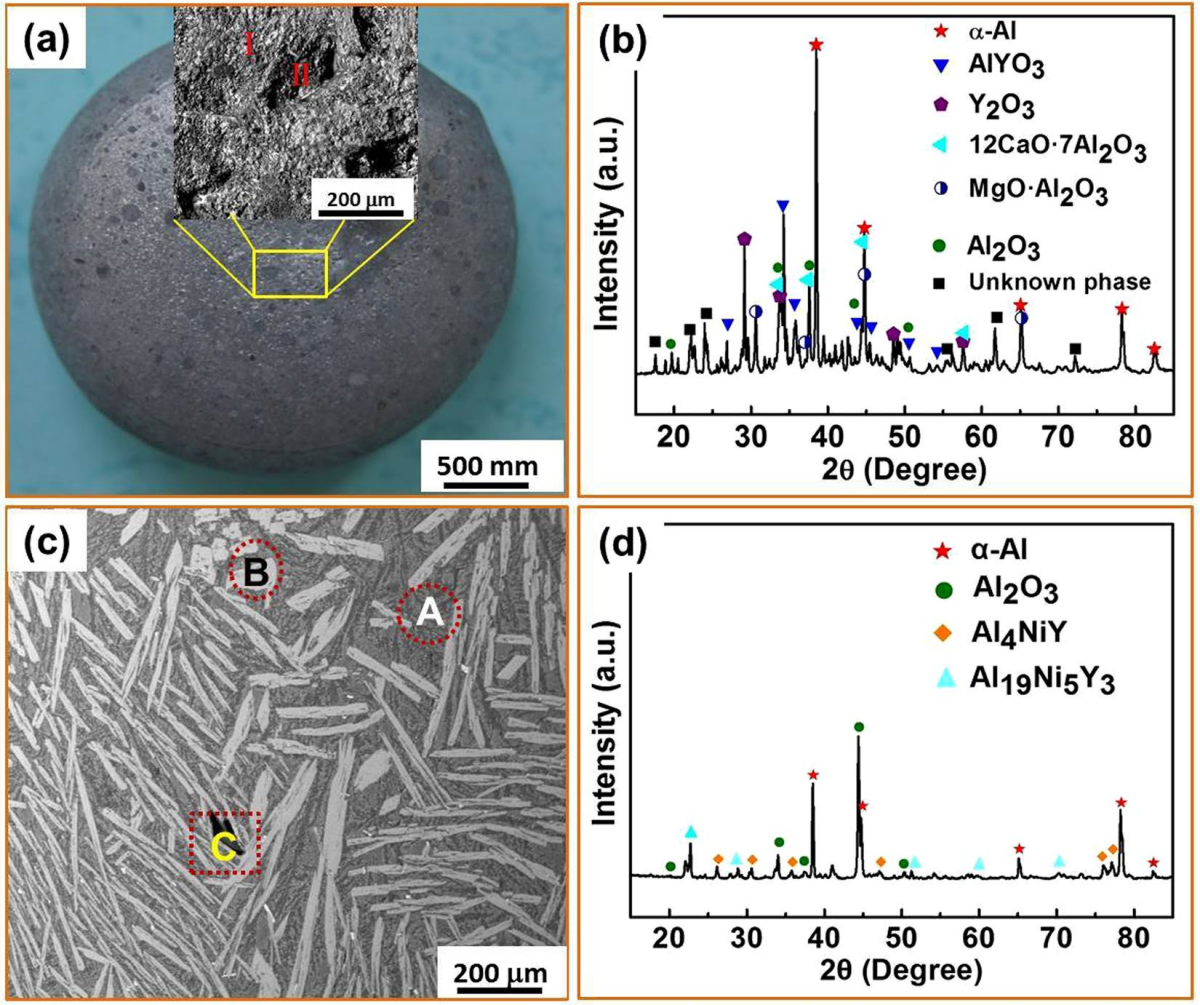
Microstructure of the Al_86_Ni_6.75_Co_2.25_Y_3.25_La_1.75_ master alloy after undergoing five-cycles fluxing treatment. (**a**) SEM image of the sample surface with an enlarged image taken from the yellow box region, showing smooth surface layer. (**b**) XRD pattern from the surface. (**c**) SEM image in typcial regions (**A**, **B** and **C**). (**d**) XRD pattern from the cross section.

To further improve the purification, we added more cycles of salt fluxing treatment. Figure 4 shows the structure of the master Al_86_Ni_6.75_Co_2.25_Y_3.25_La_1.75_ alloy after five-times MgCl_2_-CaCl_2_ salt fluxing. With the increase of fluxing time, the surface of the master alloy appears much smoother and denser since the thickness of the oxide layer on surface is gradually decreased and eventually close to that of the matrix when compared to Fig. 2a, although there is no marked change in the oxygen content. Some AlYO_3_ was found in the surface, while other phases are the same as before in the XRD scans (see Figs 3(d) and 4(d)). Moreover, Fig. 4(c,d) indicate that, in comparison with the three-times treated sample, the sizes of the dispersed second phases are further refined and the oxygen contents in the regions of the matrix and precipitated phases are reduced. In the purification treatment process, the oxide inclusions inside in the melt are continuously collected by the salts that float upwards, adding to the thickening oxygen-enriched surface. Thus, the amount of oxygen in the melt is reduced, although there is no significant change in proportion of oxide inside the surface layer.

**Figure 5:**
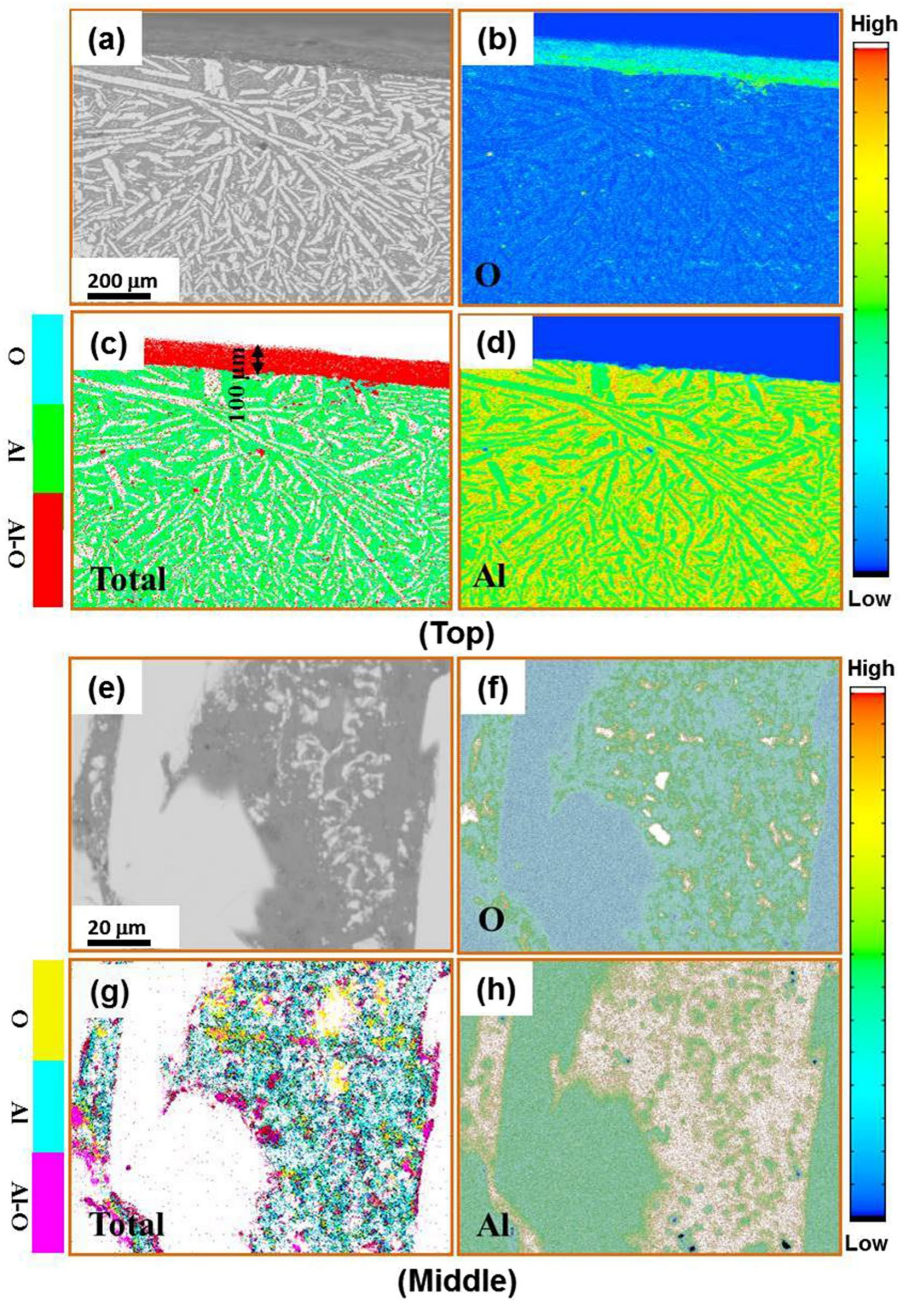
Cross-sectional EPMA mappings of the Al_86_Ni_6.75_Co_2.25_Y_3.25_La_1.75_ master alloy without fluxing treatment, showing the elemental distributions of O and Al as well as total amount on top region (**a**–**d**) and middle region (**e**–**h**), respectively.

To map out the distribution of various oxygen and salt elements in the Al-based matrix, EPMA was used after different numbers of thermal cycles. The elemental distributions of O and Al in the matrix of untreated sample, in the surface and the middle section, are displayed in Fig. 5 (see the different BSE contrasts and atomic concentrations). We see that the surface was O-enriched, and this layer was approximately 100 μm thick (Fig. 5c). The EPMA results suggest that the composition is close to Al_2_O_3_. In the middle section of the sample, O atoms were mainly in the matrix rather than in the second phase (Fig. 5f). Comparing Fig. 5b and f, the O-enriched region corresponds to reduced Al content, and the atomic ratio of Al to O was about 41.2: 59.8, which should correspond to the ~Al_2_O_3_ oxide.

**Figure 6:**
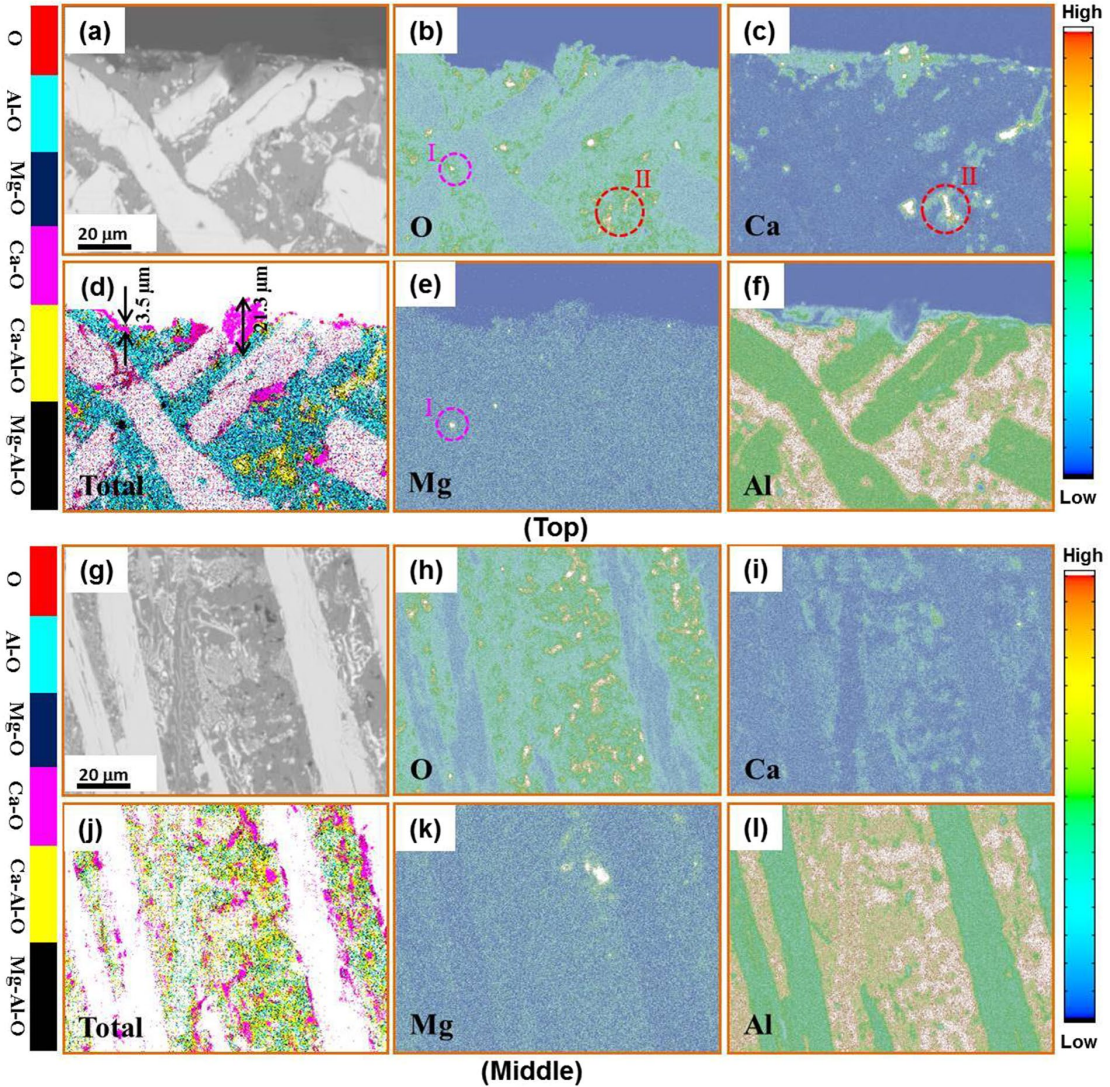
Cross-sectional EPMA mappings of the Al_86_Ni_6.75_Co_2.25_Y_3.25_La_1.75_ master alloy for three heating-cooling cycles, showing the elemental distributions of O (**b**,**h**), Ca (**c**,**i**), Mg (**e**,**k**), Al (**f**,**l**) and the total synthesis of map elements (**d**,**j**) in the sample top region (**a**–**f**) and middle region (**g**–**l**), respectively.

Figure 6 shows the EPMA mapped elemental distributions in the Al_86_Ni_6.75_Co_2.25_Y_3.25_La_1.75_ alloy after three cycles of MgCl_2_-CaCl_2_ salt fluxing treatment. On the surface region (Fig. 6a~f) and in the middle section (Fig. 6g~l) of the ingot, the Ca-enriched and Mg-enriched regions formed around the O-enriched regions (marked by I and II in Fig. 6b,c,e), which verifies the adsorption effect on oxides of the molten salt. From the total elemental analysis of EPMA, one notes that there exist different oxide enriched areas, e.g. Mg-O, Ca-O, Mg-Al-O and Ca-Al-O, respectively (see Fig. 6d and j). It is reasonable to interpret that the molten salt reacts with the oxide inclusion, forming purification products such as MgO, CaO and CaO(MgO)·Al_2_O_3_ clathrates. This is consistent with the formation of clathrates in Figs 3b and 4b. In addition, one can note that the surface film layer formed is uneven in thickness (see Fig. 6d). The main reason is that the molten salt is still reacting with a large number of oxides remaining in the middle region and could not completely float to the surface. This indicates that the purification is inadequate after three cycles.

**Figure 7:**
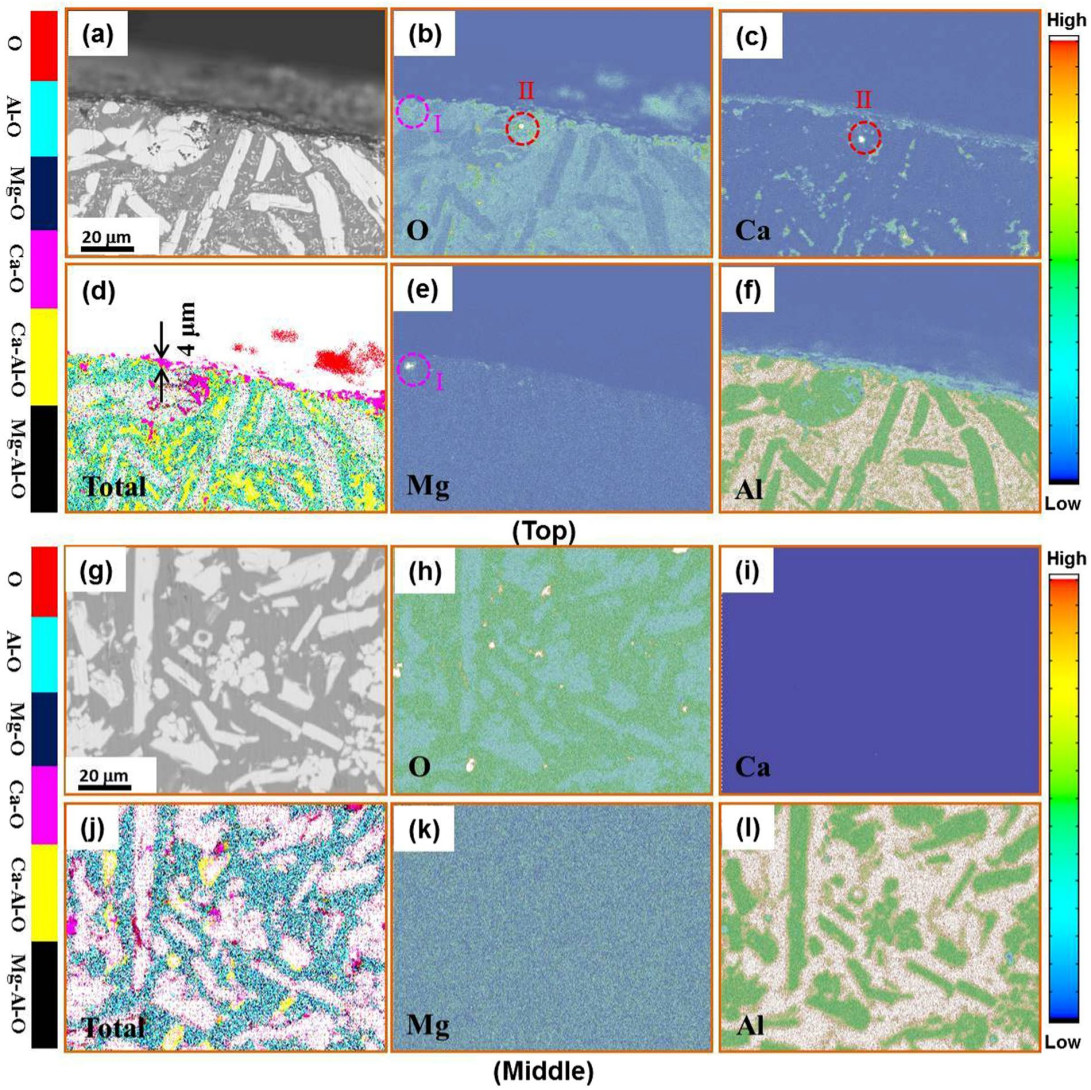
Cross-sectional EPMA mappings of the Al_86_Ni_6.75_Co_2.25_Y_3.25_La_1.75_ master alloy for five heating-cooling cycles, presenting the elemental distributions of O (**b**,**h**), Ca (**c**,**i**), Mg (**e**,**k**), Al (**f**,**l**) and the total synthesis of map elements (**d**,**j**) in the sample top region (**a**–**f**) and middle region (**g**–**l**), respectively.

The results of five-cycle treated specimen are shown in Fig. 7. Comparing Fig. 7 with Fig. 6, note that the oxidation products are mainly concentrated on the surface of the alloy (Fig. 7d) with a much reduced thickness of about 4 μm. Clearly, the oxygen distribution both in the surface film layer and in the middle section of the ingot was significantly decreased. As for the Ca and Mg elements, they are mainly located at the surface of the ingot (Fig. 7c and e), with almost no residues existing in the melt (Fig. 7i and k). This indicates that melt purification reaction is by and large completed after 5 cycles.

**Figure 8:**
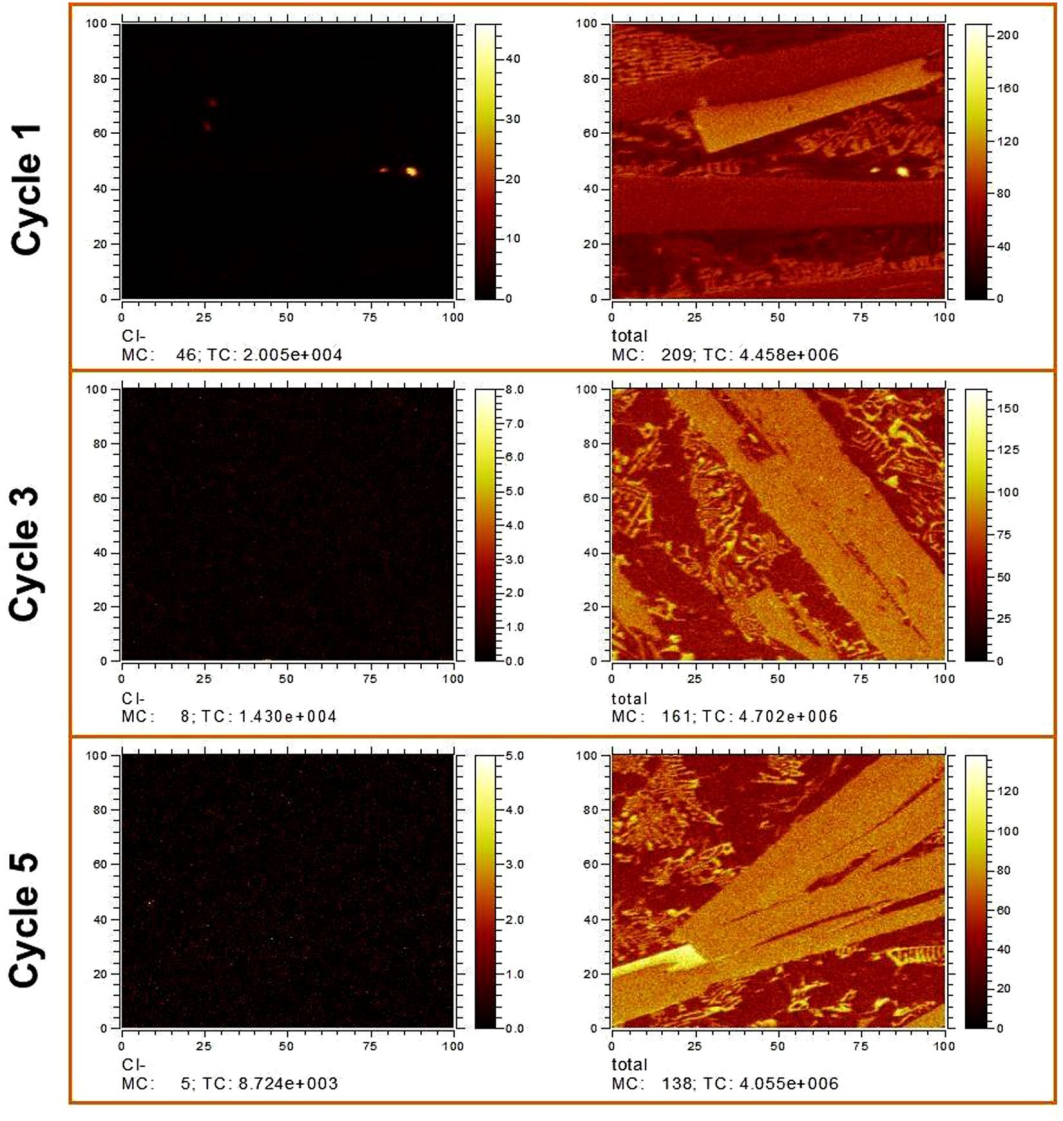
The Cl ions distribution of the alloy under different heat-cooling cycles using SIMS analysis.

**Figure 9:**
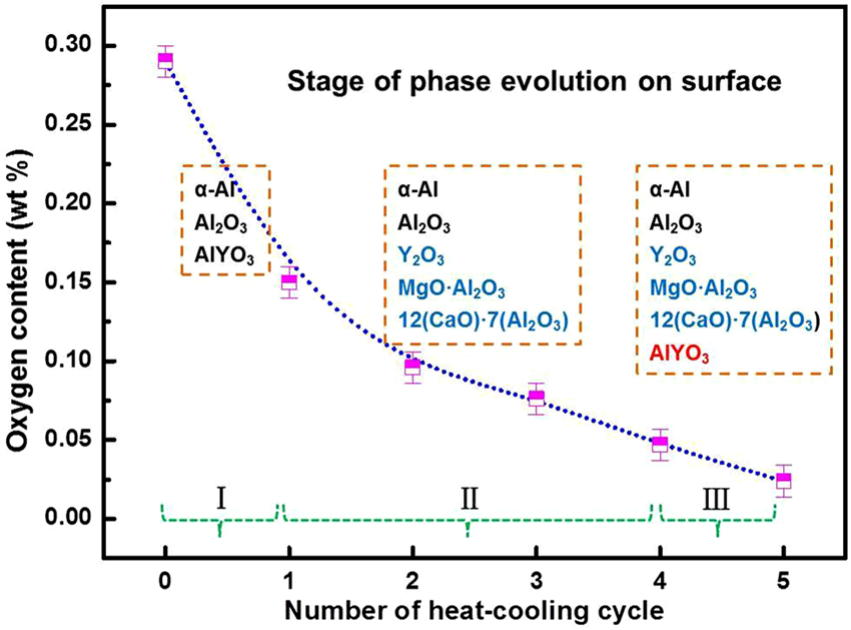
Oxygen content in the Al_86_Ni_6.75_Co_2.25_Y_3.25_La_1.75_ master alloy after different number of cycles of fluxing treatment, and corresponding phases present in the surface layer.

The above analysis indicates that the Ca and Mg cation can react with O in the melt and float to the surface of the sample. This would not affect the GFA of the Al alloy, as the surface (oxide-ridden) layer was cut off via mechanical polishing after fluxing. As for the residual Cl anions, TOF-SIMS analysis was performed to enable high sensitivity mapping of the distribution of Cl, as shown in Fig. 8 after different number of fluxing cycles. At the beginning of the purification process, Cl ions are obvious in some local regions due to the slow gas (the AlCl_3_ from the reaction MgCl_2_ + Al_2_O_3_ = MgO + AlCl_3_) discharge rate from the melt. But after three cycles, there is no aggregated Cl visible (see Fig. 8b,c). Figure 9 compares the overall oxygen content in the as-cast bulk Al_86_Ni_6.75_Co_2.25_Y_3.25_La_1.75_ master alloy sample after different cycle times. The oxygen content exhibits a monotonously decreasing trend, reducing from 0.28 wt.% for the untreated sample to 0.024 wt.% for the five-cycled sample. This also demonstrates that the fluxing treatment using the composite chlorate salt is effective in reducing oxygen from the Al-rich alloys through the removal of oxide inclusions in the melt.

### Effects of fluxing on GFA

**Figure 10:**
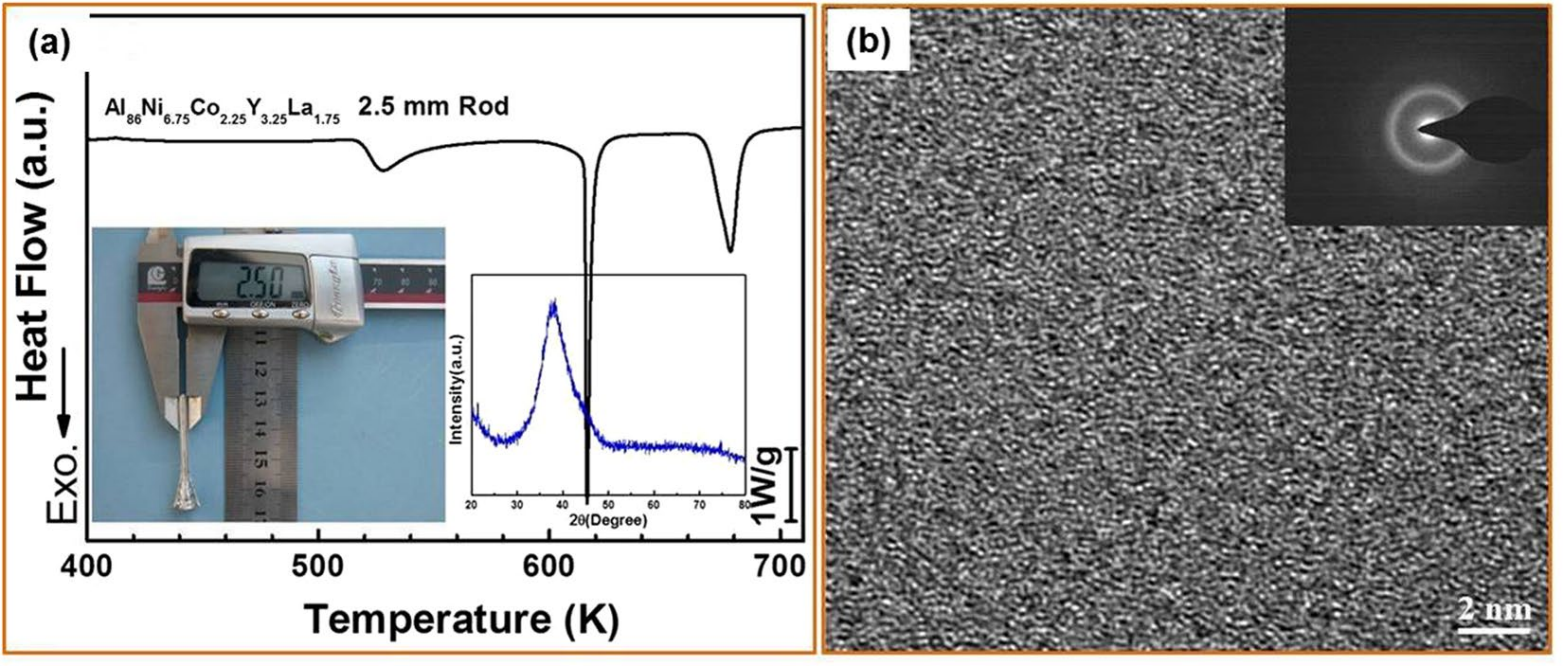
(**a**) DSC scan of the Al_86_Ni_6.75_Co_2.25_Y_3.25_La_1.75_ BMG with a diameter of 2.5 mm. The insets show the rod sample and its XRD pattern. (**b**) High-resolution TEM image and the corresponding SAED pattern (inset). The sample was taken from the center of the 2.5 mm as-cast rod.

**Figure 11:**
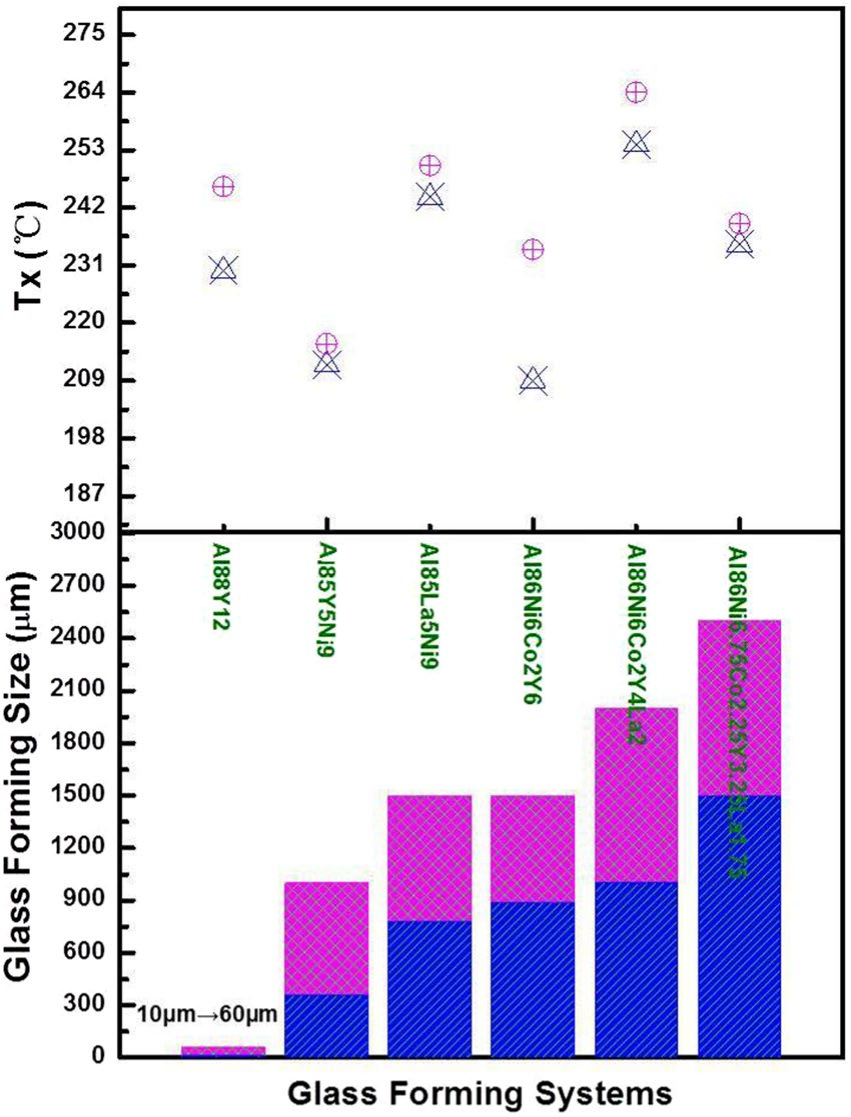
(**a**) Onset crystallization temperature and (**b**) Critical diameter of MG formation for typical Al-rich binary, ternary, quaternary and quinary alloys (i.e., Al_88_Y_12_, Al_86_Ni_9_Y_5_, Al_86_La_9_Y_5_, Al_86_Ni_6_Co_2_Y_6_, Al_86_Ni_6_Co_2_La_2_Y_4_ and Al_86_Ni_6.75_Co_2.25_La_3.25_Y_1.75_), after five cycles of fluxing treatment, compared with those (blue) with no fluxing treatment.

We now proceed to check the effect of melt-fluxing treatment on the GFA of Al-rich alloys. Without the fluxing treatment for the Al_86_Ni_6.75_Co_2.25_Y_3.25_La_1.75_ master alloy, the largest BMG reached was 1.5 mm via copper mold casting^9^. After this quinary master alloy underwent five-times fluxing treatment, the GFA has been elevated significantly: a BMG 2.5 mm in diameter has been successfully obtained. Figure 10(a) displays the outer appearance of the Al_86_Ni_6.75_Co_2.25_Y_3.25_La_1.75_ BMG sample and the amorphous characteristics in the XRD spectra. The high-resolution TEM image and the selected-area electron diffraction (SAED) pattern of this alloy are shown in Fig. 10(b). The sample was taken from the center of the 2.5 mm as-cast rod. No crystals can be detected in the high-resolution TEM image, which exhibits uniform amorphous structure. The broad halo in the SAED pattern confirms that it is monolithic amorphous phase, which agrees well with the fully amorphous feature from the XRD result. To further evaluate the effectiveness of such a composite salt treatment in elevating GFA of Al-rich alloy systems in general, we have also selected a group of typical Al-rich alloys from binary to quaternary systems. The improvement of the critical size after the fluxing treatment is at least 30%, at the best GFA compositions of these systems, as presented in Fig. 11(a). In addition, note that the thermal stability of these Al-rich MGs is also enhanced to various extents due to the fluxing. This is manifested in the increase of crystallization temperature *T*_x_ of these alloys (see Fig. 11b). Such a phenomenon is believed to be associated with the removal of oxygen impurities from the melt during the fluxing treatment, which suppresses heterogeneous crystal nucleation and improves the resistance against crystallization. The effects of residual chlorine in the melt on the forming ability of Al-BMGs remain to be a subject of future study.

## Discussion

### Mechanisms to remove Al2O3

As discussed in Section 2, there are two considerations about the selection of MgCl_2_-CaCl_2_ composite fluxing salt. Firstly, it can effectively absorb oxide impurities. Secondly, it can react with oxide impurities and form new types of oxides that easily flow to the melt surface. From the results of Figs 3(b) and 4(b) that underwent different treatment cycles, we note the formation of two clathrate products, MgO·Al_2_O_3_ and 12(CaO)·7(Al_2_O_3_), which are absent in the untreated sample in Fig. 2(b). This finding leads us to believe that chemical reaction has taken place between the MgCl_2_-CaCl_2_ molten salt and oxide inclusions in the molten alloy. The occurrence of such a reaction could be described by two key steps: (i) a replacement reaction happened between Mg ion or Ca ion and Al_2_O_3_ in the melt, *i.e*., 3[Mg]^2+^ + Al_2_O_3_ = 3MgO + 2[Al] or 3[Ca]^2+^ + Al_2_O_3_ = 3CaO + 2[Al]; (ii) owing to the physical absorption characteristics of newly-generated oxide (*e.g*. MgO or CaO), there was subsequent clathrate reaction between the MgO or CaO oxide and the residual Al_2_O_3_, *i.e*. MgO + Al_2_O_3_ = MgO·Al_2_O_3_, and 12CaO + 7Al_2_O_3_ = 12(CaO)·7(Al_2_O_3_). As such, a large amount of Al_2_O_3_ is replaced and/or clathrated. This is manifested by the appearance of MgO·Al_2_O_3_ and 12(CaO)·7(Al_2_O_3_) on the surface of the master alloy.

Interestingly, from the XRD results in Figs 3(d) and 4(d), we find no clathrate products preserved in the interior of the master alloy. This can be understood from the standpoint of migration kinetics of clathrate products. It has been recognized that the migration of clathrate products from the interior of the melt to the surface of the melt is a key process to accomplish the melt purification in Al-rich alloys. The above migration (*i.e*. floating to melt surface) requires a sufficiently fast migration rate of these fluxing-formed products. Assuming that these products are spherical particles, their ability to float up to melt surface mainly depends upon the balance of gravity, buoyancy and the fluid resistance of aluminum alloys. Correspondingly, the floating rate, *U*, of these products in the molten melt in the vertical direction could be expressed as^64^:

6 $$U=2{R}^{2}({\rho }_{1}-{\rho }_{p})g/9\mu $$

where *R* is the radius of the products, *ρ*_l_ and *ρ*_p_ are the densities of Al alloy liquid and the products, respectively, *g* is acceleration of gravity, and *μ* is viscosity coefficient of the Al alloy melt. From Eq. (6), the floating rate of the products is proportional to the square of the radius of the products and the difference in density between the products and melt, but inversely proportional to the viscosity coefficient of the melt. That is to say, the larger the product size is and the smaller the liquid viscosity of aluminum alloy is, the faster the floating velocity becomes. Normally, for the clathrate product such as 12(CaO)•7(Al_2_O_3_), the radius is about 100 μm and the density is 2.83 g/cm3, the viscosity coefficient, *μ*, is lower than 1.5 N•s/m2, and the density of Al melt is about 3.1 g/cm^3^ in Al-based multicomponent system. So the floating rate of clathrate products calculated from Eq. (6) is approximately 4 × 10^−3^ cm/s. The distance from bottom to surface is usually 1 cm for our master alloy, thus the overall time of clathrate products moving from the interior of melt to the surface of melt is calculated to be less than 5 min., which is the time period of our normal salt-treatment per cycle. This indicates that after the salt-treatment of 3 or 5 cycles, the clathrate products formed during chemical reaction would have adequate time to arrive at the surface of the master alloy.

**Figure 12:**
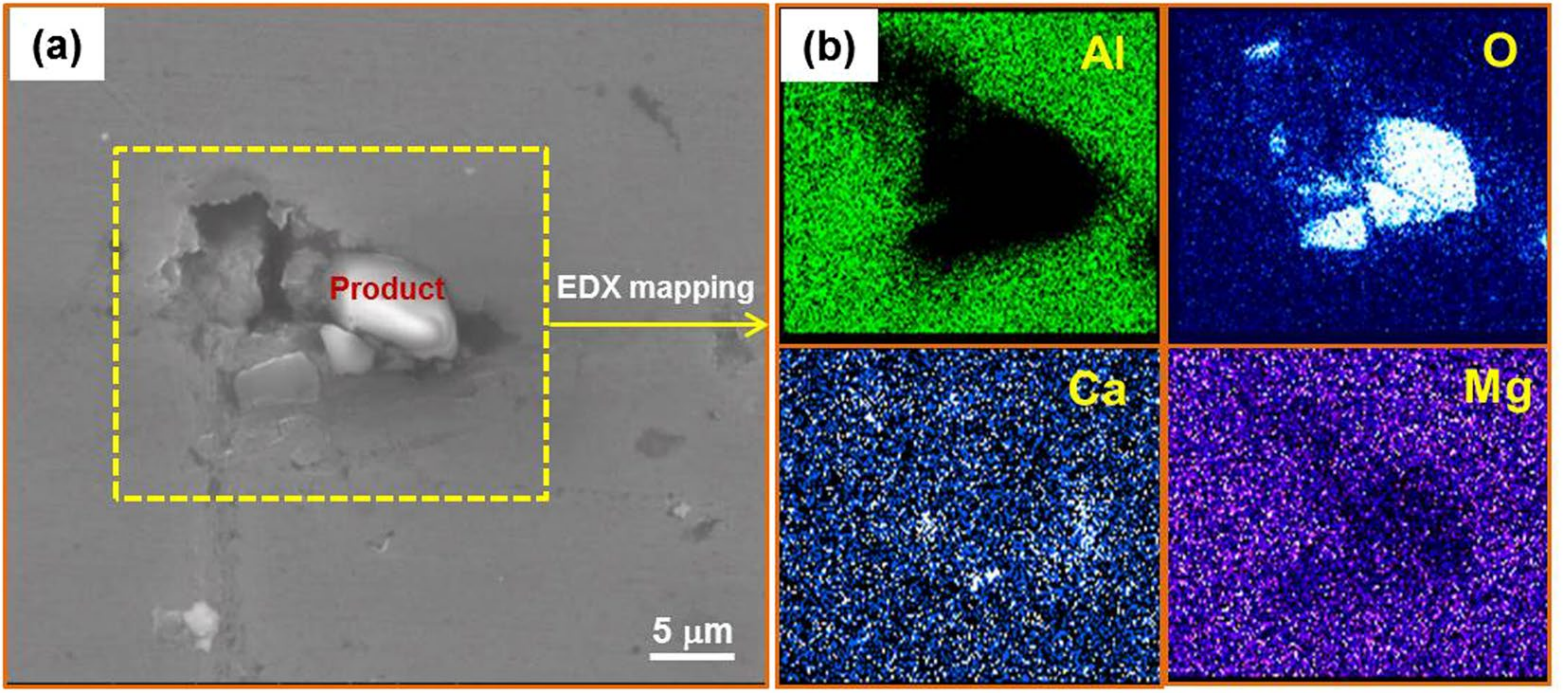
SEM image of Al solidified after an early stage of fluxing treatment of the melt. (**a**) Al_2_O_3_ inside Al (**b**) EDX mapping of the yellow boxed region in (**a**).

The complex products seen in Figs 3(b) and 4(b) on the surface of the fluxing-treated master alloy makes it difficult to clarify exactly the physical absorption effect of the salt on Al_2_O_3_ during melt purification. To confirm the occurrence of physical absorption, we used a simple melt of pure aluminum to investigate the fluxing mechanism of MgCl_2_-CaCl_2_ salt. Considering that physical absorption normally takes place prior to chemical reaction in fluxing process, we mainly focused on the initial stage of salt-fluxing Al_2_O_3_ particles in pure Al melt. Figure 12(a) displays a typical region containing Al_2_O_3_ particles. From the SEM image on this region in Fig. 12(b), we note that Al_2_O_3_ particles appeared around the defects located in this region. And magnesium and calcium elements distributed around the oxide although a small amount of calcium elements is infiltrated into the oxide. This suggests that the Ca/Mg salts and Al_2_O_3_ tend to segregate together and enclose one another, which verifies that physical absorption indeed played a role in the purification. The above experimental results are what expected from the ideas behind our design of the salt agents.

### The role of rare earth oxides

Along with the melt purification (see Figs 2 (b), 3(b) and 4(b)), we also find that rare earth oxides formed on the surface. They evolve from AlYO_3_ (untreated) → Y_2_O_3_ (three-times treatment) → AlYO_3_+Y_2_O_3_ (five-times treatment), as marked in Fig. 10. This phenomenon could be understood on the basis of a thermodynamic analysis in the Y-Al-O system^65^. In general, yttrium has a stronger affinity with oxygen than other elements in Al-TM-RE systems and yttrium oxide (Y_2_O_3_) is more thermodynamically stable. However, the initial oxidation product is AlYO_3_ rather than Y_2_O_3_ in the outmost part of the untreated master alloy. This is because Y reacts with oxygen to form Y_2_O_3_ oxide within the melt, followed by reaction with the Al melt:

7 $${{\rm{Y}}}_{2}{{\rm{O}}}_{3}+[{\rm{Al}}]\to {{\rm{AlYO}}}_{3}+[{\rm{Y}}].$$

The brackets denote that Al and Y are in the liquid solution. According to thermodynamic data^66, 67^, the equilibrium constant, *K*, is equal to 6.5 × 10^−4^ at 1423 K. Thus, the formation of AlYO_3_ in the melt could take place if the ratio of activity, *a*_Y_/*a*_Al_, is less than 6.5 × 10^−4^. In the equilibrium conditions, this ratio is constant at a certain temperature. However, the activities of Y and Al in equilibrium state and their concentrations could be changed when the composite chlorate salt is added into the melt and reacted with Al_2_O_3_. Correspondingly, the heat absorbed by this reaction process could result in a decrease of temperature, and the equilibrium conditions of Y and Al activities in Eq. (7) no longer hold. Thus, Y_2_O_3_ appeared in the middle of fluxing (*i.e*. salt-treatment with 1~4 cycles). Further, we can expect that, when the added fluxing salt has reacted with Y_2_O_3_ phase completely after five-cycles, it would no longer hinder the occurrence of the reaction in Eq. (7) and the AlYO_3_ phase reappeared.

This study demonstrates the beneficial fluxing effects of chloride salts on the GFA of Al-TM-RE metallic glass-forming melts. We have designed fluxing salts based on combined physical absorption (the interfacial tension) and chemical reaction/absorption (between the salt and oxides) effects: the present MgCl_2_-CaCl_2_ composite salt takes advantage of both. Our experimental data showed obviously decreased oxygen content due to fluxing treatment, from 0.28 wt% to 0.024 wt% for the bulk sample. After the master alloy was subjected to five cycles of fluxing treatment at the Al_86_Ni_6.75_Co_2.25_Y_3.25_La_1.75_ composition, a record GFA was achieved, increasing the critical size for the formation of fully amorphous BMG rod from 1.5 mm to 2.5 mm in diameter. This degree of GFA elevation is similar to those reported previously for successful B_2_O_3_ fluxing treatment in Pd- and Fe- based BMG-forming systems^68, 69^. The fluxing effects investigated in this work are also of general interest, and particularly informative for reaching desired GFA in other reactive alloy systems.

## Methods

### Chloride salts synthesis and fluxing treatment

The chloride salts (MgCl_2_ and CaCl_2_) selected are commercially available anhydrous material with the purity of 99.9 wt.%. The two chloride salts were mixed together in proportion uniformly, put into a dry and cleaned ceramic crucible (9.0 cm for inner diameter and 23.5 cm for height), and then heated up to 400 °C for 5 min to remove some contaminants from the mixture. Next, the partially mixed salts were further heated up to their melting point, kept for 10 min under 1 MPa pressure, and subsequently cooled down in a resistance-heated furnace under a vacuum of 1.0 × 10^−1^ Pa. The mixture finally was cut into pieces and then put into the blender mixer for 10 hours to fine the mixture powders.

**Figure 13:**
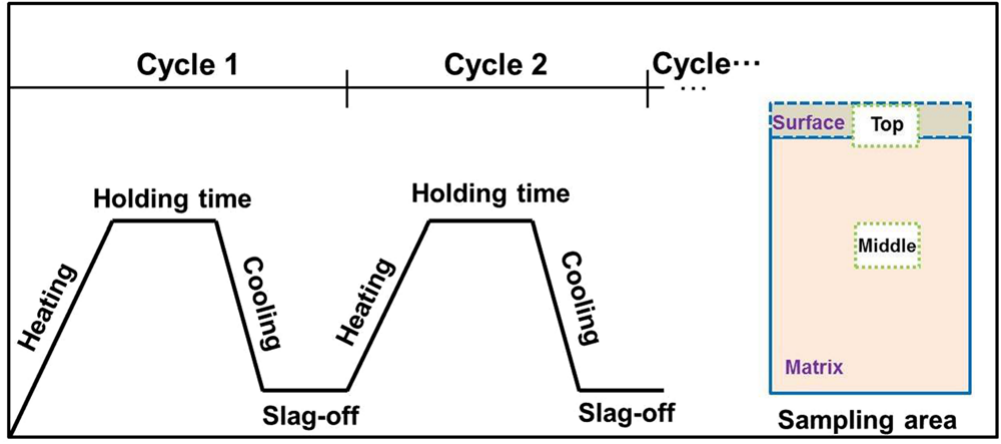
Schematic illustration of the salt-fluxing process with the same heat-cooling cycles, the interior picture is the sampling and analysis area under different processing.

For the Al-TM-RE alloy melts, elemental pieces with purity better than 99.99 wt.% were used as starting materials. A series of Al-TM-RE master alloy ingots with the nominal composition (in at.%) were prepared by arc melting under a Ti-gettered argon atmosphere in a water-cooled copper crucible. The alloy ingots were melted six times to ensure compositional homogeneity. Rod-shaped samples were obtained by the injection of the molten alloy into the cavity of a copper mold with a diameter of 1 mm and 2.5 mm. During the fluxing experiment, the MgCl_2_ and CaCl_2_ composite salts and an Al_86_Ni_6.75_Co_2.25_Y_3.25_La_1.75_ alloy ingot were put together into a dry and cleaned water cooling copper crucible, and then subjected to heating-cooling thermal-cycles treatment in an electric-arc furnace under a vacuum of 5 × 10^−3^ Pa. The master alloy and the salts were heated up to about 1000 °C and held for 4 min and then cooled to room temperature together with the crucible. The detail illustration of the process is shown in Fig. 13. The mixture salts initially placed at the bottom of the ingot in order to ensure that the molten salt fully enters and captures the oxide in the melt. In this way, the captured oxide is eventually concentrated on the surface of the alloy and can then be removed by mechanical polishing. For a more effective fluxing treatment, such a heating-cooling cycle was repeated several times.

### Microstructural characterization

To evaluate the effects of chloride salts on melt purification and the GFA, detailed microstructural analyses were performed on the master alloy surface as well as the cross-sectional surfaces of the master alloy and cast rod sample via x-ray diffraction (XRD) using a Rigaku D/max 2400 diffractometer (Tokyo, Japan) with monochromated Cu Kα radiation (λ = 0.1542 nm). The oxygen content in the samples before and after molten-salt fluxing treatment was monitored using a TC600 LECO oxygen analyzer. Three samples were taken from each ingot, after different purification process (number of cycles). To further detect the microstructural evolutions associated with fluxing treatment, Scanning electron microscopy (SEM) and transmission electron microscopy (TEM) images were taken for the samples prior to, in the middle of, and after purification using FEI Quanta 600 and FEI Tecnai F30 electron microscope, respectively. The chemical mapping of the alloying elements and the oxides, across cross-section of samples, was carried out using an electron probe microanalyser (EPMA, SHIMADZU 1720H, with an accelerating voltage of 25 kV and a beam current of 100 nA). Second ion mass spectroscopy (SIMS) analysis was performed using a ToF-SIMS V instrument (ION-ToF, GmbH, Münster, Germany) equipped with a 25 keV Bismuth LMIG (Liquid metal ion gun) and an 10 keV Cs sputter gun, enabling high sensitivity of Cl to observe its distribution as a function of depth. The as-cast rods were also analyzed using differential scanning calorimetry (DSC) in a Perkin-Elmer DSC7 at a heating rate of 20 K/min.
